# Comparative Analysis of Electrostatic Models for Ligand Docking

**DOI:** 10.3389/fmolb.2019.00052

**Published:** 2019-07-03

**Authors:** Geraldo Rodrigues Sartori, Alessandro S. Nascimento

**Affiliations:** São Carlos Institute of Physics, University of São Paulo, São Carlos, Brazil

**Keywords:** ligand docking, polar interactions, electrostatic energy, Poisson-Boltzmann, Coulomb

## Abstract

The precise modeling of molecular interactions remains an important goal among molecular modeling techniques. Some of the challenges in the field include the precise definition of a Hamiltonian for biomolecular systems, together with precise parameters derived from Molecular Mechanics Force Fields, for example. The problem is even more challenging when interaction energies from different species are computed, such as the interaction energy involving a ligand and a protein, given that small differences must be computed from large energies. Here we evaluated the effects of the electrostatic model for ligand binding energy evaluation in the context of ligand docking. For this purpose, a classical Coulomb potential with distance-dependent dielectrics was compared with a Poisson-Boltzmann (PB) model for electrostatic potential computation, based on DelPhi calculations. We found that, although the electrostatic energies were highly correlated for the Coulomb and PB models, the ligand pose and the enrichment of actual ligands against decoy compounds, were improved when binding energies were computed using PB as compared to the Coulomb model. We observed that the electrostatic energies computed with the Coulomb model were, on average, ten times larger than the energies computed with the PB model, suggesting a strong overestimation of the polar interactions in the Coulomb model. We also found that a slightly smoothed Lennard-Jones potential combined with the PB model resulted in a good compromise between ligand sampling and energetic scoring.

## Introduction

The quantitative description of molecular interactions, at an atomic level, remains an important challenge even in current days of *Petascale* computing. Some of the difficulties found in this field include: (i) the energetic description of biomolecular systems; (ii) the fact that binding energies are small differences taken from large energies, resulting in large uncertainties; and (iii) the limited sampling for some calculations. Taken together, these obstacles are exactly the challenge of scoring solutions in the docking problem (Halperin et al., 2002).

The second problem, due to the small differences taken from bigger numbers, can be alleviated with accurate calculations and appropriate sampling. In the context of single point calculations, such as in ligand docking, this challenge remains as an important issue and is handled in some applications with a posterior analysis of ligand candidates using molecular dynamics (MD) or Monte Carlo (MC) simulations to generate an ensemble of thermally accessible configurations of the system and binding energy calculations. In this context, the MM-GBSA or MM-PBSA approaches became very popular (Graves et al., 2008; Zhang et al., 2014; Genheden and Ryde, 2015).

The energetic description of a biomolecular system is tackled in many docking approaches using molecular mechanics force fields (Luty et al., 1995; Schulz-Gasch and Stahl, 2004; Cisneros et al., 2014), where the intermolecular interaction energies are typically computed as a sum of polar interactions, modeled as a Coulomb potential, and van der Waals interactions, modeled through a Lennard-Jones potential (Wang and Lin, 2013). Additional terms can be added to model the influence of the solvent, for example (Muniz and Nascimento, 2017).

Modeling polar interactions using a Coulomb potential introduces some potentially important issues. First, polarization is not considered. Although this effect might be important, a quantum description of the system would be required for appropriate treatment of the dynamics in the electron density within the active site, increasing the computational costs of the calculation. Polarization could also be taken into account by the use of polarizable force fields. However, the computational cost associated with these calculations limits their use in the context of the docking of large compound databases (Illingworth et al., 2008). Second, the dielectric medium of a protein might not be exactly a constant medium, since the protein surface faces the solvent while its core might be closer to a highly hydrophobic medium. So, a representation of the electrostatic potential (and energies) as a function of a varying continuum dielectrics might be necessary, such as the treatment given by the Poisson-Boltzmann (PB) equation (Honig and Nicholls, 1995; Oron et al., 2003; Li et al., 2012, 2013).

Interestingly, Luty et al. observed that, for 20 poses of benzamidine within 8 Å of trypsin binding site, the electrostatic interaction energy computed with PB and using a simple Coulomb model assuming ε = *f*(*r*), i.e., the dielectric constant ε is a linear function of the interatomic distance *r*, showed a high correlation (*r*^2^ = 0.96) (Luty et al., 1995). In contrary, Gilson and Honig observed that this simple distance-dependent dielectric model overestimates electrostatic interactions (also observed by Luty et al.) and concluded that this model does not seem to be a realistic way of treating polar interactions in biomolecular systems (Gilson and Honig, 1988).

In late '80s Honig et al. developed the DelPhi program (Gilson et al., 1988), that numerically solves the PB equation for macromolecular structures, of any shape, given atomic coordinates, atomic van der Waals parameters, and atomic charges. The calculation of electrostatic potentials within the current versions of Delphi (Li et al., 2012, 2013; Jia et al., 2017) is fast, taking a few seconds in typical workstation computers for a small size protein. Although it might not be fast enough to be used in MD simulations, it is very competitive for docking studies, where the receptor is kept as rigid, in many strategies, and the interaction potentials can be pre-computed in grids and stored for the actual docking calculations (Meng et al., 1992; Luty et al., 1995). Worth of note, the PB calculation is under constant improvement. Recently, Li et al. showed that Gaussian-based smoothed dielectric function could better reproduce the assignment of PKa's for protein residues (Li et al., 2013). The same approach was also applied to the ion distribution (Jia et al., 2017).

Here, we compared the results of docking enrichments and pose reproduction within the same algorithm when using Coulomb electrostatics with a distance-dependent dielectric model (i.e., ε = *r*) and using a PB electrostatic potential pre-computed using DelPhi (Li et al., 2012). Concurrently, we evaluated the influence of Lennard-Jones soft-core potential on docking efficacy with both PB and Coulomb models. We found that the PB electrostatic model resulted in modest improvement in pose reproduction and enrichment. However, when this model was combined with a smoothed van der Waals potential, an important improvement of pose reproduction and enrichment was observed, suggesting that fine-tuning of these terms is necessary.

## Materials and Methods

### Docking Calculations

For all docking calculations reported in this work, the software LiBELa (dos Santos Muniz and Nascimento, 2015) was used. LiBELa (Ligand Binding Energy Landscape) uses a combination of ligand- and receptor-based strategies for docking. For this purpose, the algorithm requires a reference ligand, that indicates the initial binding mode. The docking procedure starts with a superposition of the *search* ligand onto the *reference* ligand by using a ligand-based approach, as previously described (Vaz de Lima and Nascimento, 2013). Briefly, LiBELa describes the volume of each *i* ligand atom as a Gaussian function (Vaz de Lima and Nascimento, 2013):

$$\begin{array}{l} \rho \left(r\right)=\;p_{i}\exp^{\left\{\left[-\pi {\left(\frac{3p_{i}}{4\pi \sigma_{i}^{3}}\right)}^{\frac{2}{3}}\right]{\left(r-r_{i}\right)}^{2}\right\}} \end{array}$$

where p_*i*_ is the Gaussian amplitude, defined as $2\sqrt{2}$, r_*i*_ is atomic coordinate for atom *i* and σ_*i*_ is the van der Waals radius for the same atom. Using this Gaussian-based description of shape, an overlay volume for two molecules, A and B, can be defined as (Vaz de Lima and Nascimento, 2013):

$$\begin{array}{l} V_{AB}=w\underset{i\;\in \;A}{\sum }\underset{j\;\in \;B}{\sum }\int dr\;\rho_{i}\left(r\right)\rho_{j}\left(r\right) \end{array}$$

Similar terms are added to V_AB_ to compute for the superposition of atoms with positive charge and negative charge with weights defined by *w* (here, set to 1.0 for both terms). Thus, by maximizing the overlay volume V_AB_ in Cartesian space, an initial optimized placement of the search ligand is obtained. Afterward, this initial binding mode is re-optimized to find a minimum in the binding energy using a global optimization algorithm. In this step, a typical force field-based definition of binding energy is used as the objective function:

$$\begin{array}{l} E_{bind}=\;\overset{rec}{\underset{i}{\sum }}\overset{lig}{\underset{j}{\sum }}\frac{q_{i}q_{j}}{\epsilon r_{ij}}+\frac{A_{ij}}{r_{ij}^{12}}-\frac{B_{ij}}{r_{ij}^{6}} \end{array}$$

Where *q* is atomic charge, r_*ij*_ is the interatomic distance between atoms *i* and *j* and A_*ij*_ and B_*ij*_ are the Lennard-Jones parameters for the atom pair *ij*, computed by the geometric mean approximation. Here, $A_{i}=2\delta_{i}{\left(2r_{0}\right)}^{12}$ and $B_{i}=2\delta_{i}{\left(2r_{0}\right)}^{6}$, where *r*_0_ is the atomic radius and δ is the well depth parameter necessary for computing van der Waals interactions according to AMBER force field. Both parameters are taken from AMBER FF14SB force field (Maier et al., 2015). A final similarity index can be computed using a Hodgkin's similarity index (Hodgkin and Richards, 1987) defined as:

$$\begin{array}{l} SI=\;\frac{2V_{AB}}{V_{AA}+V_{BB}} \end{array}$$

To speed up the calculations, the receptor interaction potential is pre-computed and stored in grids. In this point, LiBELA can compute a typical Coulomb electrostatic potential:

$$\begin{array}{l} \phi \left(r\right)=\;\overset{rec}{\underset{i}{\sum }}\frac{q_{i}}{\varepsilon r_{i}} \end{array}$$

where the dielectric constant was set to the interatomic distance *r*, i.e., ϵ = *r*_*ij*_ (Luty et al., 1995). Alternatively, LiBELa can parse a DelPhi electrostatic map with the electrostatic potential ϕ_DelPhi_ instead and compute binding energies using this stronger electrostatic model. For the calculations shown in this work, DelPhi 8.4 was used (Li et al., 2012, 2013). Typically, a computation box of 30 × 30 × 30 Å with a spacing of 0.4 Å (gsize 75 and scale 2.5 Å, in Delphi parameters), with interior dielectrics of 2.06 and exterior dielectrics of 78.5, and salt concentration set to 145 mM. The same grid spacing was used in calculation employing the Coulomb model.

We also tested the effect of a smoothed Lennard-Jones potential by applying the same strategy as suggested by Verkhivker et al. (1999). Here, the binding energy is evaluated as (dos Santos Muniz and Nascimento, 2015; Muniz and Nascimento, 2017):

$$\begin{array}{l} E_{bind}=\overset{lig}{\underset{j}{\sum }}q_{j}\phi \left(r\right)\overset{rec}{\underset{i}{\sum }}\overset{lig}{\underset{j}{\sum }}\frac{A_{ij}}{{\left(r_{ij}^{6}+\delta_{VDW}^{6}\right)}^{2}}-\frac{B_{ij}}{r_{ij}^{6}+\delta_{VDW}^{6}} \end{array}$$

The smoothing term δ_VDW_ was systematically varied in the interval 0.5 to 2.5 Å with a step of 0.5 Å to evaluate the effect of the Lennard-Joned soft-core potential in pose reproduction and enrichment when combined with a Coulomb electrostatic potential (ϕ_Coulomb_) or a PB electrostatic potential (ϕ_Delphi_).

### Docking Pose Reproduction

#### Self-Docking Test

For docking pose reproduction, we used three data sets. The dataset SB2012 (Mukherjee et al., 2010) includes 1,043 crystal structures of protein-ligand complexes, distributed as SYBYL MOL2 files. Here and all over this text, the “receptor” is defined as a protein where an organic small molecular, the “ligand.,” binds. In these files, the atomic charges are already defined using AMBER forcefield for receptor and AM1-BCC (Jakalian et al., 2000, 2002) for ligands. The dataset files were used as provided, with no further optimizations or modifications of atomic coordinates. Here, a docking calculation was set using each ligand-receptor pair, using the own ligand as the reference ligand in LiBELa.

#### Cross-Docking Test

For a cross-docking experiment, the Astex dataset was used (Verdonk et al., 2008). In this dataset, 58 structures with analogous complexes are provided. From this dataset, 54 targets were used together with 860 ligands in total. The targets (receptors) were prepared using DockPrep tool as available in UCSF Chimera (Pettersen et al., 2004) using AMBER FF14SB atomic charges. For the ligands, AM1-BCC atomic charges were attributed using ANTECHAMBER (Wang et al., 2006) and SYBYL atom types were assigned using the same tool. In this experiment, each ligand was docked on different (non-native) crystal structures of its own target. Afterward, the root mean square deviation (RMSD) was computed using the native ligand structure as a reference.

### Enrichment Tests

In order to evaluate the ability to enrich actual ligands against decoys, i.e., compounds with similar physicochemical properties but not expected to bind to a given target, the DUD38 subset of DUD-E database, which contains 38 targets from the original DUD dataset (Huang et al., 2006), but rebuild with the same protocol as used in DUD-Enhanced (DUD-E) (Mysinger et al., 2012). This subset includes the PDB files for the receptors and over 630,000 compounds, among binders and decoys, with an average decoy-to-ligand ratio of 33. The compounds were used as provided (as SYBYL MOL2 files) with atomic charges defined following the default ZINC protocol (Irwin and Shoichet, 2005; Irwin et al., 2012). The receptor files were prepared using the DockPrep tool available in UCSF Chimera (Pettersen et al., 2004). In this tool, atomic charges are attributed to receptor atoms following AMBER FF14SB parameters. Finally, the prepared receptor is saved as a SYBYL MOL2 file type.

The target-specific ligands and decoys were docked to each target using LiBELa default parameters and using either a Coulomb electrostatic model or a pre-computed Delphi electrostatic potential. The Delphi calculations were carried out in two steps. In the first step, a calculation is set where the protein represents 50% of the calculation box. In a second step, a focused calculation was carried out using a grid of 0.4 Å for a 30 × 30 × 30 Å calculation box centered in the center of mass of the reference ligand. The energies computed after docking calculations were used to rank the docked molecules and ROC curves were computed with locally developed python scripts. The enrichment was quantified using the Adjusted LogAUC metric (Mysinger and Shoichet, 2010). This metric is similar to the well-known AUC but is computed for a semi-logarithmic plot of the ROC curve spanning three decades in the horizontal axis. The computed area is then corrected to remove the area expected for a random enrichment (14.5%).

## Results

The calculations of the electrostatic potentials with DelPhi are very fast, typically taking <5 s in an Intel Xeon E5645 (2.40 GHz) processor running in a single thread. This is much faster than the calculation of the interaction potential grids in LiBELa, which took about 5.4 min averaging over the 38 targets of the DUD38 dataset. The computational efficiency of the electrostatic calculations with DelPhi makes it tempting to use this more robust model in docking calculations. However, what is the actual role of PB-based calculation on protein-ligand interactions in the context of ligand docking? In order to address this question, we set up a comparative analysis of ligand binding poses and ligand enrichments using the Coulomb electrostatic model or PB as the electrostatic model to assess the effect of the model in sampling and enrichment, respectively.

### Effect on Sampling

The simplest experiment one can think of to evaluate the sampling effect on ligand docking is to assess the ability of a model to reproduce ligand poses from crystal structures. In this context, we compared the RMSDs observed for LiBELa using either Coulomb or PB as the electrostatic model.

After the redocking of 1,029 ligands on their respective receptors, the RMSD for all atoms, including hydrogen atoms, was computed in comparison with the original (experimental) structures. Averaging over the entire dataset an RMSD of 1.215 Å was observed for the Coulomb model, while for the PB model an average RMSD of 1.129 Å was achieved. The median RMSD for these models were 0.535 and 0.598 Å with a standard deviation slightly increased for Coulomb as compared to PB (1.689, compared to 1.419 Å). For both models the fraction of targets with RMSD values found below the typical cutoff value of 3.0 Å was close to 90%, as indicated in Table 1.

**Table 1:** Summary of the self-docking experiment using the SB2012 dataset.

**Electrostatic model**	**Coulomb**	**PB**
**Smoothing parameter** **δ_VDW_** **=** **0.0 Å**		
Average RMSD (Å)	1.215	1.129
Median RMSD (Å)	0.535	0.598
Standard deviation for RMSD (Å)	1.689	1.419
RMSD < 3.0 Å (%)	90%	92%
Average HA_RMSD (Å)	1.124	1.035
Average HA_RMSDh (Å)	1.035	0.948
Average HA_RMSDm (Å)	0.534	0.494
Average Hodgkin's similarity index	0.858	0.871
**Smoothing Parameter** **δ_VDW_** **=** **0.5 Å**		
Average RMSD (Å)	1.287	1.079
Median RMSD (Å)	0.535	0.596
Standard deviation for RMSD (Å)	1.812	1.312
< 3.0 Å (%)	88%	93%
Average HA_RMSD (Å)	1.192	0.987
Average HA_RMSDh (Å)	1.104	0.901
Average HA_RMSDm (Å)	0.538	0.467
Average Hodgkin's similarity index	0.852	0.875
**Smoothing Parameter** **δ_VDW_** **=** **2.0 Å**		
Average RMSD (Å)	5.643	1.185
Median RMSD (Å)	5.566	0.611
Standard deviation for RMSD (Å)	2.304	1.525
< 3.0 Å	15%	91%
Average HA_RMSD (Å)	5.356	1.096
Average HA_RMSDh (Å)	4.881	1.007
Average HA_RMSDm (Å)	2.263	0.527
Average Hodgkin's similarity index	0.287	0.858

*N = 1,030. Statistics for RMSDs computed after redocking of ligands are shown in comparison with experimental poses. HA_RMSD, HA_RMSDh and HA_RMSDm refer, respectively, to the average standard RMSD computed for heavy-atoms only, the minimum-distance heavy-atom RMSD used in Autodock Vina (Trott and Olson, 2009) and Hungarian (symmetry-corrected) heavy-atom RMSD (Kuhn, 2010), as computed by DOCK6 (Brozell et al., 2012)*.

When a smoothed Lennard-Jones potential was combined with the electrostatic models under evaluation in this work, we found very interesting differences. For a small smoothing parameter δ = 0.5 Å, the differences between the electrostatic models are small, similarly to what is observed in the AMBER Lennard-Jones model. However, as δ becomes larger, the differences between the Coulomb model and the PB model become more evident. When δ is set to 2.0 Å, the average RMSD found for the Coulomb model was 5.643 Å (median 5.566 Å), while the average RMSD for the PB model was 1.185 Å, with median in 0.611 Å. So, it appears that the combination of the PB model with a soft-core VDW potential still leads to good results in pose reproduction while the Coulomb model rapidly seems to dominate the binding energy resulting in meaningless ligand poses.

**Figure 1:**
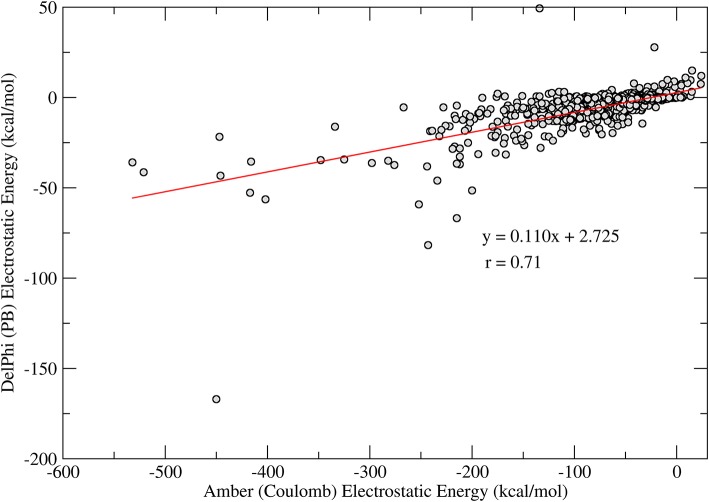
Correlation of the electrostatic interaction energies computed with Coulomb model (horizontal axis) and PB (vertical axis). The line shows a linear regression of the obtained data (*N* = 1,029) and the regression coefficients are shown in the figure.

Another interesting observation comes from the comparison between the polar term in the interaction energies. An analysis for 1,029 protein-ligand complexes reveals a good correlation between the electrostatic interaction energies computed using a Coulomb model with distance-dependent dielectrics, i.e., ε = r_*ij*_, and electrostatic interaction energies computed using the Poisson-Boltzmann model. As shown in Figure 1, there is a good correlation between the computed energy terms (*r* = 0.7 for *N* = 1,029), as also observed previously by Luty et al. (1995). Additionally, one can observe that the electrostatic interaction energies computed by the Coulomb model are about 10 times more favorable, on average than those computed using the PB model, indicating a typical overestimation of the interaction energies in this model. In the context of ligand docking, this overestimation may result in binding modes that are biased toward a few polar contacts that are too favorable as compared to the overall fitting of the ligand and receptor binding pockets.

A more stringent test is to assess the ability of the model to reproduce experimentally determined poses in a cross-docking experiment, i.e., in a receptor structure different from the one used in the actual docking calculation. In brief, it involves docking ligand L_A_ in receptor structure R_B_ and comparing the docking pose to the pose observed when L_A_ was crystallized bound to receptor R_A_. For this task, the Astex non-diverse dataset was used.

The employed dataset includes 603 diverse (non-native) complexes. The results obtained are summarized in Table 2. Again, when the typical AMBER Lennard-Jones potential is used, a slight improvement in the binding poses is observed, with average RMSD going from 4.48 Å, for the Coulomb model, to 3.95 Å in the PB model (median values of 3.27 and 2.94 Å, respectively). When a smoothed Lennard-Jones potential is used, on the other hand, the differences between both electrostatic models increase. For a smoothing parameter δ set to 0.5 Å, the average RMSD decreases from 4.65 in the Coulomb model to 3.90 in the PB model (median values 3.52 and 3.06 Å). And when δ is set to 2.0 Å, the average RMSD decreases from 7.60 to 3.71 Å, with median values decreasing from 7.52 to 2.54 Å (Table 2).

**Table 2:** Summary of the cross-docking experiment using the Astex dataset.

**Electrostatic model**	**Coulomb**	**PB**
**Smoothing parameter** **δ_VDW_** **=** **0.0 Å**		
Average RMSD (Å)	4.477	3.951
Median RMSD (Å)	3.269	2.938
Standard deviation for RMSD (Å)	4.408	4.091
Average HA_RMSD (Å)	4.194	3.698
Average HA_RMSDh (Å)	3.768	3.272
Average HA_RMSDm (Å)	4.075	3.801
Average Hodgkin's similarity index	0.533	0.562
**Smoothing parameter** **δ_VDW_** **=** **0.5 Å**		
Average RMSD (Å)	4.653	3.900
Median RMSD (Å)	3.523	3.064
Standard deviation for RMSD (Å)	4.537	4.014
Average HA_RMSD (Å)	4.362	3.635
Average HA_RMSDh (Å)	3.851	3.270
Average HA_RMSDm (Å)	4.074	3.790
Average Hodgkin's similarity index	0.521	0.562
**Smoothing parameter** **δ_VDW_** **=** **2.0 Å**		
Average (Å)	7.603	3.712
Median (Å)	7.519	2.542
Standard deviation (Å)	3.758	4.053
Average HA_RMSD (Å)	7.369	3.457
Average HA_RMSDh (Å)	6.721	3.077
Average HA_RMSDm (Å)	5.699	3.688
Average Hodgkin's similarity index	0.206	0.579

*N = 603. Statistics for RMSDs computed after redocking of ligands in a non-native receptor structure are shown in comparison with experimental poses*.

Taken together, the results shown here indicate that the PB model for electrostatic computation result in better pose reproduction in the scenario of a typical AMBER FF binding energy calculation and, more significantly, in the scenario of a soft docking, i.e., when the Lennard-Jones potential is smoothed. Given the results obtained, we moved for the evaluation of the changes in the enrichment of actual binders against decoy compounds.

### Ligand Enrichment

In order to assess the ability of the electrostatic models to recover actual ligands against decoy compounds, we choose the DUD38 dataset. In this dataset, 38 targets are given with a set of binder compounds and a set of decoy compounds. In this context, a decoy is defined as a compound that has similar physicochemical properties to the binders but is not expected to bind to the receptor. After docking all the binders and decoys, the compounds are ranked by their binding energy and a receiver-operating characteristic (ROC) curve is plotted. Finally, the enrichment is computed using Adjusted LogAUC metric, as previously proposed (Mysinger and Shoichet, 2010). Briefly, this metric describes the area under the curve of the ROC plot with the *x*-axis in the logarithm scale and spanning three decades. The area computed is corrected by subtracting the area expected for a random enrichment.

The results obtained are summarized in Table 3 and shown in the complete version in the Supplementary Material. From the data shown here, we note that, for the usual Lennard-Jones model used in AMBER force field, i.e., δ_VDW_ = 0.0, the electrostatic models performed almost similarly in terms of enrichment, with an average enrichment of 4.50 or 4.91 for Coulomb or PB, respectively, with a slight improvement of the enrichment with the PB model. Using the smoothed VDW potential with δ_VDW_ = 0.5 Å, similar enrichments are observed but with an improvement in the median enrichment for the PB model. Here, the average enrichments were 5.01 and 4.99 with median enrichments of 3.93 and 5.40 for Coulomb and PB models, respectively. Finally increasing the smoothing constant to δ_VDW_ = 2.0 Å, a maximum in the average/median logAUC is observed for the PB model (5.36 and 6.09 for average and median, respectively), while a marked decrease in the enrichment for the Coulomb model is observed.

**Table 3:** Summary of the enrichment experiment using the DUD38 dataset.

**Electrostatic model**	**Coulomb**	**PB**
**Smoothing parameter** **δ_VDW_** **=** **0.0 Å**		
Average	4.50 (56.2%)	4.91 (57.3%)
Median	3.24 (54.9%)	4.70 (56%)
Standard deviation	6.87 (10.4%)	4.42 (8.3%)
**Smoothing parameter** **δ_VDW_** **=** **0.5 Å**		
Average	5.01 (57.1%)	4.99 (57.5%)
Median	3.93 (56.4%)	5.40 (57.2%)
Standard deviation	6.81 (10.5%)	4.03 (7.7%)
**Smoothing parameter** **δ_VDW_** **=** **2.0 Å**		
Average	1.80 (53.4%)	5.36 (58.7%)
Median	0.52 (52.2%)	6.09 (58.6%)
Standard deviation	5.14 (8.5%)	3.87 (7.4%)
**Dock 6.7 Grid Score**		
Average	1.3 (42.5%)	
Median	−1.2 (43.3%)	
Standard deviation	9.7 (17.4%)	

*The values are reported as Adjusted LogAUC and also as AUC, in the parenthesis*.

For the sake of comparison, the same docking calculations using the DUD38 were set up using the Grid Score model of DOCK 6.7 (Brozell et al., 2012). The average and median logAUC observed for this model was 1.3 and −1.2, respectively (Table 3). Since logAUC corrects for the expected random enrichment, this metric can achieve negative results if results are worse than random. It is important to add that the Grid Score used here has a 6–12 Lennard-Jones potential with a Coulomb electrostatic model that uses a distance-depend dielectric function (ε = r_*ij*_), similar to the model used in LiBELa.

## Discussion

The enrichment data shown in Table 3 for the DUD38 dataset strongly suggests that the continuum electrostatic model can lead to important improvements in the ability to recover actual binders and separate them from decoy compounds. On the other hand, as we already noted from the data shown in Figure 1, the Coulomb interaction electrostatic energies are, on average, 10 times more favorable than interaction electrostatic energies computed with PB. Then, it makes sense that the balance between the electrostatic and van der Waals terms should be also fine-tuned. We assessed this balance by introducing a smoothed Lennard-Jones term to model the van der Waals interactions. A good balance seems to be achieved when the smoothing constant δ_VDW_ was set to 2.0 Å. With this calculation setup, a maximum in the enrichment is observed, without compromising the docking poses, according to the results of ligand enrichment with DUD38, self-docking with the SB2012 dataset (Table 1) and cross-docking with the Astex dataset (Table 2). A complete comparison of the effect of the smoothing parameter δ_VDW_ is shown in the Supplementary Material, where the ligand pose and ligand enrichment can be compared as a function of the smoothing parameter.

A second effect of the electrostatic treatment given to the docking calculations can be observed in the distribution of the net charges of the top-scored molecules in docking calculations. The analysis of the charge distribution for the target ace, shown as an example in Supplementary Figure 2, reveals that among the top-scored molecules when the Coulomb model was used, almost half of them have net charges −2 or −3 *e*, indicating a favoring of the non-specific electrostatic interactions to the total docking score. On the other hand, the PB model favors neutral molecules or molecules with net charge −1 *e* (Supplementary Material). No molecule with net charge −2 or −3 *e* is observed among the top-scored molecules, suggesting a much more specific scoring of the biomolecular interactions. As a piece of evidence of the correctness of the PB model, an inspection of the distribution of net charges among the actual binders in the DUD dataset for this target shows that 66% of the binders have net charge 0, 30% have charge −1 and 4% have charge −2, indicating that the PB model more closely reflects the molecular interactions observed in experimental conditions.

A recent development in the PB calculations introduced a Gaussian-based approach “to deliver a smooth dielectric function for the entire space domain” (Li et al., 2013). The authors showed that the Gaussian-based function resulted in better assignments of PKa's and also dielectric values for protein interior and protein-water interface in agreement with previous works (Li et al., 2013). Interestingly, a comparison of the ligand enrichment obtained after docking calculations using the Non-Gaussian dielectric model and the Gaussian dielectric model showed a decrease of about 15% in the enrichment of actual ligands against decoys in the DUD38 dataset. This decrease is observed for the AMBER Lennard-Jones model as well as for the smoothed Lennard-Jones model. This still preliminary observation highlights that even the robust PB model can still be improved in the context of ligand docking to result in even more reliable calculations and predictions of protein-ligand interactions.

What is the actual role of PB calculations in protein-ligand recognition? A simpler answer to this question would be a better treatment of intermolecular electrostatic interactions avoiding the overestimation of charge-charge interaction, as observed in the Coulomb model. However, as observed by Basu et al. (2012), there is a close association between electrostatic complementarity and shape complementarity in biological macromolecules. This close association may be also associated with the findings shown here, where tradeoff between a better electrostatic treatment for intermolecular interactions and adjustment of van der Waals interactions results in better recognition model, directly affecting sampling (better binding poses for ligands) as well as scoring (better enrichment of known ligands when compared to decoy compounds).

In conclusion, here we evaluated the effect of scoring docking calculations with a Coulomb model or with a Poisson-Boltzmann model for electrostatic energies. As the major findings, we observe that (i) PB model improves both docking pose and docking scoring capabilities. In the most stringent test for pose reproducibility, the cross-docking test, the best results were found for the combination of PB and softcore Lennard-Jones potential. Here we observed a reduction of the average RMSD from 3.95 to 3.90 Å and then to 3.71 Å, as the softcore constant δ goes from 0.0 to 0.5 Å and then to 2.0 Å. (ii) The best enrichment results for the challenging dataset DUD38 was also observed for the combination of PB and softcore Lennard-Jones potential. Again, taking the median values over the DUD38 dataset, we observed an increase of 15 or 30% if δ_VDW_ is set to 0.5 or 2.0, respectively. These results confirm previous observations that the Coulomb potential overestimate the electrostatic energy in protein-ligand interaction calculations and also show that a re-parametrization of the Lennard-Jones parameters might be necessary. Finally, given the quick calculations of PB-based electrostatic potentials in modern workstations, we don't see a reason for not using this model in protein-ligand calculations in the context of ligand docking.

## Data Availability

The datasets generated for this study are available on request to the corresponding author.

## Author Contributions

AN conceived the project. GS conducted the experiments. GS and AN collected and analyzed the data. All authors wrote and approved the manuscript.

### Conflict of Interest Statement

The authors declare that the research was conducted in the absence of any commercial or financial relationships that could be construed as a potential conflict of interest.
